# Structural Stability of Sulfur-Depleted MoS_2_

**DOI:** 10.1021/acsnanoscienceau.5c00172

**Published:** 2026-02-11

**Authors:** Ygor M. Jaques, Cristiano F. Woellner, Lucas M. Sassi, Marcelo L. Pereira Junior, Luiz A. Ribeiro Junior, Pulickel M. Ajayan, Douglas S. Galvão

**Affiliations:** † Group of Organic Solids and New Materials (GSONM), Gleb Wataghin Institute of Physics, 28132 University of Campinas (UNICAMP), Campinas, SP 13083-859, Brazil; ‡ Center for Computational Engineering & Sciences (CCES), University of Campinas (UNICAMP), Campinas, SP 13083-970, Brazil; § Department of Physics, 28122 Federal University of Paraná (UFPR), Curitiba, PR 81531-980, Brazil; ∥ Department of Materials Science and NanoEngineering, 3990 Rice University, Houston, Texas 77005, United States; ⊥ College of Technology, Department of Electrical Engineering, 28127 University of Brasília, Brasília, DF 70910-900, Brazil; # Computational Materials Laboratory (LCCMat), Institute of Physics, University of Brasília, Brasília, DF 70910-900, Brazil; ¶ Interdisciplinary Center for Science, Technology, and Innovation (CICTI), Federal University of Paraná (UFPR), Curitiba, PR 81530-000, Brazil

**Keywords:** MoS_2_, sulfur depletion, defect reconstruction, molecular dynamics, transition metal dichalcogenides, surface migration

## Abstract

Transition metal dichalcogenides (TMDs), particularly monolayer MoS_2_, have received increased attention in materials science and have been exploited in diverse applications, from photonics to catalysis. Defects in TMDs play a crucial role in modulating their properties, and understanding defect-induced dynamics is of great importance. This study investigates the dynamics of sulfur depletion in defective monolayer MoS_2_, which yields stable MoS monolayers. Various defect sizes, temperature regimes (300–1000 K), and substrate effects were investigated. Through comprehensive classical molecular dynamics (CMD) and *ab initio* molecular dynamics (AIMD) simulations, we elucidate the dynamics of sulfur vacancy formation in MoS_2_ lattices. After removal of all sulfur atoms from the top layer, several sulfur atoms from the bottom layer spontaneously migrate to the top layer as a response to increase structural stability, thus creating a MoS_*x*_ alloy. These findings deepen our understanding of defect dynamics in TMDs, offering valuable insights into the controlled engineering of their properties for nanotechnology applications.

## Graphical abstract

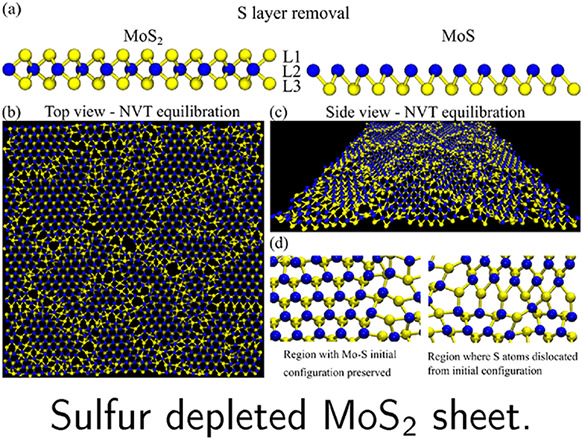

## 1 Introduction

Transition metal dichalcogenides (TMDs) have recently received significant attention due to their attractive properties, impacting various research fields, including photonics, plasmonics, valleytronics, catalysis, optoelectronics, and flexible electronics.^1–11^ Monolayer TMDs consist of three atomic layers: a hexagonally close-packed transitional metal layer sandwiched between two chalcogen layers, forming an MX_2_-type compound (M = Mo, W, Sn, Nb, Ta; X = S, Se, Te).^12,13^ Among the two-dimensional (2D) materials family, MoS_2_ is of particular interest, having been the object of several studies due to its electrical,^14^ optical,^15^ and other physical properties, from bulk to monolayer forms.^16,17^

Defects in the structure of these materials can significantly impact their properties.^18^ First-principles and experimental studies suggest that S-vacancies often introduce donor-like states and that Mo vacancies tend to introduce acceptor-like states.^19^ The effective doping observed in devices, however, is strongly influenced by extrinsic factors, such as unintentional impurities, surface adsorbates, contact effects, and Fermi-level pinning. As a result, the doping character associated with individual defect types remains an open topic in the literature.^20–22^ In the case of MoS_2_, sulfur vacancies induce n-doping, while molybdenum ones induce p-type behavior.^19^ Structural defects have also been shown to affect mobility and device performance significantly.^23,24^ Generally, changes in the electronic structure caused by defects lead to changes in excitonic processes, thereby modifying the optical properties. For instance, the photoluminescence (PL) spectrum of MoSe_2_ exhibits shifts in its peaks with the creation of Se vacancies.^25^ On the other hand, new peaks appear in the PL spectrum of MoS_2_ when bisulfur vacancies are created through α particle bombardment.^26^

Proton irradiation of MoS_2_ samples has been found to induce ferromagnetic behavior, likely due to the generation of vacancies and the contribution from defective zigzag or armchair edges that create surface states.^27^ Consequently, it is crucial to create defects in a controlled manner to tailor the material properties. Methods for achieving this control include bombardment with argon ions,^28^ He ions,^29,30^ α particles,^26^ protons,^27^ Mn^2+^ ions,^31^ exposure to oxygen plasma,^32^ and electron beam irradiation,^33^ among others. Beyond TMDs, first-principles modeling has demonstrated that the incorporation of defects and variations in chalcogen or heteroatom content can also induce diverse structural reconstructions in two-dimensional (2D) and graphene-like networks. The synthetic growth concept (SGC) predicts that specific vacancy motifs, ring rearrangements, and heteroatom incorporation, such as fluorine, can lead to the formation of fullerene-like, graphitic, or amorphous domains in CF_*x*_ films, with well-defined compositional thresholds and energetics.^34,35^

One application in which defects in TMDs play a crucial role is the hydrogen evolution reaction (HER). TMDs are well-known for their chemically inert basal planes, necessitating the introduction of defects to enhance reactivity in these materials.^36^ Theoretical calculations have revealed that sulfur vacancies in MoS_2_ expose Mo atoms and their d-orbitals, making them more reactive.^37^ Experimental studies have confirmed that S-vacancies in 2H-MoS_2_ exhibit higher activity for HER compared to edge sites or the 1T-phase.^36,38^ Furthermore, it has been demonstrated that increasing the number of vacancies enhances the reaction efficiency, and applying strain to the defective material further boosts HER activity.^39^

Highly defective TMD structures can also serve as crucial postsynthesis modifications for rationalizing 2D alloys. The literature has previously demonstrated that sulfur atoms in the top layer of MoS_2_ can be selectively sputtered by Ar^+^ ions, followed by Se substitution through Se evaporation, leading to the synthesis of the alloy MoS_2(1–*x*)_Se_2*x*_.^40^ Additionally, Ghorbani-Asl et al. discussed the possibility of synthesizing an alloy, MoSF, composed of molybdenum, sulfur, and fluorine, exhibiting metallic behavior through a similar process.^41^

In this work, we assessed the stability of a highly defective MoS_2_ structure through classical molecular dynamics (CMD) and *ab initio* molecular dynamics (AIMD) simulations. This defective lattice is obtained by systematically removing the entire top sulfur layer to examine the structural rearrangements of the resulting configuration at room temperature. Following the removal of half of the sulfur atoms, the resulting structure is afterward referred to as MoS, maintaining stoichiometric consistency. Additionally, we investigated the interaction between the equilibrated MoS layer and a pristine MoS_2_ one. Subsequently, the interaction was analyzed to investigate how it evolves with increasing temperatures, from 300 to 1000 K.

## 2 Methodology

In this study, we have carried out comprehensive fully atomistic CMD simulations, employing both CMD and AIMD approaches to investigate the behavior of defective MoS_2_ monolayers and bilayers. For the CMD simulations, we used the LAMMPS code^42^ within the framework of the reactive ReaxFF potential.^43^ Our systems encompassed a 3 × 3 × 1 hexagonal supercell for the MoS_2_ monolayer, characterized by dimensions of 9.57 × 9.57 × 43.13 Å^3^. For the larger monolayer and bilayer MoS sheets, we employed an orthorhombic simulation box measuring approximately 95.69 × 93.92 × 50 Å^3^. In this context, MoS denotes the sulfur-depleted configuration obtained after complete removal of the top chalcogen layer, MoS_*x*_ refers to partially repopulated regions where sulfur migration leads to a local stoichiometry *x* < 2, and depleted region indicates areas where the sulfur coverage remains lower than that of pristine MoS_2_ following reconstruction.

We initiated our simulations by performing structural optimization of pristine MoS_2_ systems using the conjugate gradient algorithm to minimize the total energy and residual forces. Subsequently, sulfur atoms were selectively removed, including the entire top sulfur layer, in both monolayer and bilayer configurations, in order to construct sulfur-depleted models. The resulting systems were then reoptimized to ensure full structural relaxation after atomic removal. Following this stage, CMD simulations were performed to achieve thermal and mechanical equilibration. Each system was equilibrated for 100 ps in the canonical (NVT) ensemble, followed by an additional 100 ps in the isothermal–isobaric (NPT) ensemble at temperatures of 300, 600, 800, and 1000 K, under a pressure of 0 bar.

We focus on the two temperature regimes. The first, 300 K, represents room and device operating conditions where sulfur-depleted MoS_2_ must remain structurally stable. The second, between 600 and 1000 K, encompasses postgrowth and processing conditions commonly applied to transition metal dichalcogenides, including annealing, chalcogen exchange, and precatalysis activation. These elevated temperatures accelerate thermally activated processes, enabling the observation of sulfur migration and Mo–S bond rearrangements within nanosecond-scale molecular dynamics trajectories while remaining below the threshold for rapid structural disorder. NVT and NPT simulations were performed at 300, 600, 800, and 1000 K.

In this work, stability refers to the persistence of the reconstructed MoS configurations over the molecular dynamics time scales explored, characterized by the absence of further large-scale structural degradation or spontaneous reversal of sulfur migration. This usage denotes kinetic stabilization and dynamical metastability rather than global thermodynamic stability with respect to pristine MoS_2_ or alternative sulfur-containing phases.

In the bilayer setup, we restrained S atoms at the bottom of the pristine layer with a force of 10 kcal/mol along the *z*-direction to mimic substrate effects. Temperature and pressure were controlled using a Nosé–Hoover thermostat,^44,45^ with time constants of 10 fs for temperature and 100 fs for pressure regulation. Our goal was to use a minimal, lattice-matched support that isolates depletion-driven mechanisms without introducing additional interfacial chemistry. A pristine MoS_2_ monolayer was selected because it provides a commensurate lattice registry with the overlayer, avoids the introduction of new elements or reactions, and relies on a parameter set (CMD or AIMD) that is already validated for Mo–S interactions. This choice also reflects a realistic bilayer contact frequently encountered during growth, transfer, or multilayer regions.

For the AIMD simulations, we have used the Quantum Espresso code.^46^ Our study focused on hexagonal supercells composed of 3 × 3 × 1 unit cells of monolayer MoS_2_, consistent with our previous CMD simulations. Before considering the cases involving sulfur-depleted structures, we first relaxed the pristine configurations. Electron exchange-correlation effects were treated by using the Perdew–Burke–Ernzerhof (PBE) functional within the generalized gradient approximation (GGA). The Kohn–Sham equations were solved by using the projector augmented wave (PAW) method with a plane-wave basis set and a cutoff energy of 500 Ry.

In our structural relaxation simulations, we employed a Monkhorst–Pack scheme for *k*-point sampling utilizing a 14 × 14 × 1 *k*-point mesh. However, for the AIMD calculations, we opted for a more efficient approach by using only the Γ point to save substantial computational time. We have used the Verlet integrator, allowing the simulation box to move/relax along the surface in the *x* and *y* directions while keeping the *z*-direction fixed. Structures were relaxed with convergence thresholds of 10^–4^ Ry/Bohr for atomic forces on each ion and 10^–6^ Ry for electronic relaxation. We introduced a 20 Å vacuum slab along the *z*-direction to prevent spurious interactions between periodic (mirror) images.

## 3 Results and Discussion

Figure 1a illustrates the schematic representation of the MoS_2_ monolayer configuration considered in this study. Initially, we began with a pristine MoS_2_ structure measuring approximately 10 × 10 nm^2^. One of its sulfur layers (L1) was entirely removed to create the MoS structure. Energy minimization calculations were carried out for both MoS_2_ and MoS structures, resulting in relaxed Mo–S bond lengths of approximately 0.243 and 0.279 nm, respectively. The removal of L1 in MoS_2_ led to an increase in the bond length values. Still, all remaining sulfur atoms remain in the L3 layer, indicating that this configuration is at the very least a local energy minimum.

**Figure 1:**
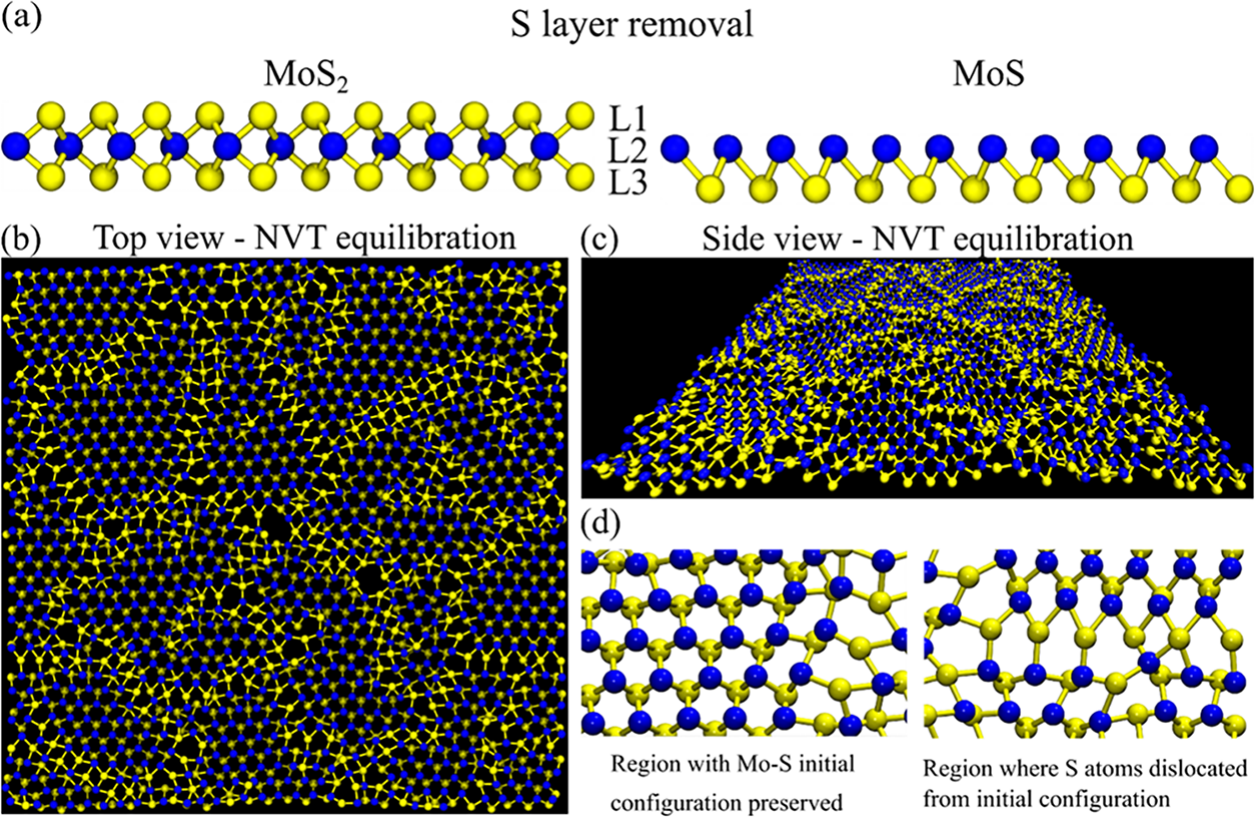
(a) A schematic representation of a MoS_2_ monolayer with one of its sulfur layers (L1) removed, leading to the formation of an MOS structure containing only one Mo layer (L2) and one S layer (L3). (b) Top and (c) side views of the equilibrated MoS configuration after 100 ps of CMD in the NVT ensemble. (d) Detailed view of two regions within the MoS structure, illustrating areas where the initial MoS configuration was preserved and areas displaying significant S dislocations.

After energy minimization, we carried out molecular dynamics simulations of MoS in an NVT ensemble at 300 K. The structure was equilibrated within 100 ps. Within approximately 10 ps, sulfur atoms began migrating to the other side of the Mo layer (L2), which, consequently, has been depleted of sulfur. Figure 1b,c illustrate that this migration was incomplete but occurred as line dislocations scattered across the surface. In the regions between these lines of sulfur atoms, it was observed that large structure areas retained the initial MoS configuration, as depicted in Figure 1d. Importantly, the Supporting Information shows CMD videos for the sulfur depletion process in defective monolayer MoS_2_. These videos capture the sulfur depletion dynamics over 20 ps in a monolayer MoS_2_ with dimensions of 100 × 100 nm^2^, containing 236,800 atoms. Supporting Videos 1–3 show different perspectives: the full view, a zoomed-in view, and a lateral zoomed-in view, respectively. In these videos, we can observe that the MoS formation still holds for large systems.

The sulfur migration fraction was evaluated by computing, for each simulation frame, the geometric center of Mo atoms along the *z*-direction, COM­(Mo, *z*), which defines the reference plane separating the upper and lower chalcogen layers. Each S atom was classified according to its vertical coordinate (*z*_S_) as below (*z*_S_ < COM­(Mo, *z*) – Δ*Z*), within (|COM­(Mo, *z*) – *z*_S_| ≤ Δ*Z*), or above (*z*_S_ > COM­(Mo, *z*) + Δ*Z*), using Δ*Z* = 0.5 Å. The choice of Δ*Z* = 0.5 Å is comparable to the typical out-of-plane thermal fluctuations of S atoms, and moderate variations of this threshold do not alter the qualitative trends or the equilibrium migration fractions. At equilibrium, approximately 18% of S atoms occupy the upper region and about 12% fluctuate within the interfacial zone at 300 K. The fraction in this intermediate region remains nearly constant at higher temperatures, while the upper fraction gradually increases, indicating that thermal activation mainly promotes out-of-plane sulfur migration across the Mo plane rather than local oscillations near it. The equilibrium distributions, obtained from the steady regime of each trajectory, showed minimal dependence on system size or ensemble choice, with differences below the statistical uncertainty.

The initial sulfur crossing event is a thermally activated process driven by the strong asymmetry created by complete sulfur removal on one side of the Mo plane. This depletion generates significant local strain and undercoordinated Mo sites, making sporadic out-of-plane sulfur displacement energetically favorable. Once a single S atom traverses the Mo plane, it partially relieves local stress and restores coordination, thereby lowering the barrier for subsequent atomic rearrangements and triggering collective reconstruction.

A similar process occurs in smaller systems, allowing us to contrast the system dynamics using the CMD and AIMD. Figure 2 presents the time evolution of a MoS structure, revealing that sulfur atoms transverse the Mo layer within a few picoseconds of dynamics, after which the system remains stable. Significantly, consistently, for both methodologies, a single S atom moves first, followed by two others. At the same time, the remaining S atoms stay at the L3 layer.

**Figure 2:**
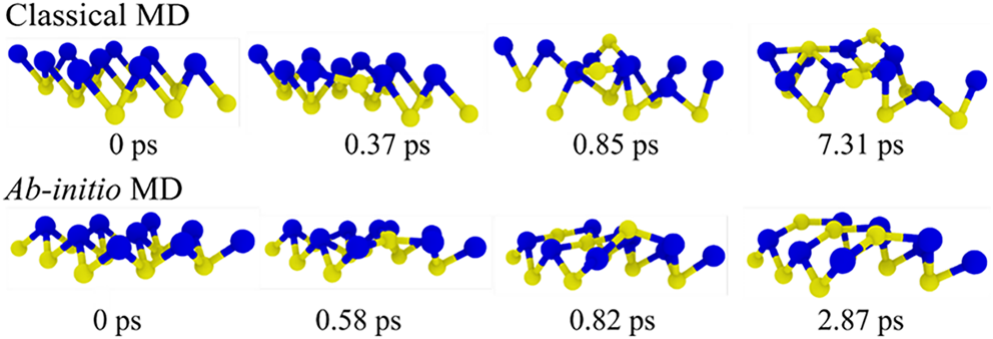
Dynamics of a 3 × 3 supercell of MoS for CMD and AIMD simulations. Both systems reached equilibrium within a few picoseconds, during which sulfur (S) atoms transverse through the Mo layer.

To confirm the system’s stability under conditions where the simulation box dimensions were allowed to change along the *x* and *y* directions (i.e., along the surface), we performed additional CMD simulations in an NPT ensemble using a larger system. Figure 3a illustrates that the monolayer undergoes a rapid structural contraction, resulting in regions where the initial Mo–S configuration is preserved but becomes partially curved. This structural shrinking process occurs within approximately 30 ps, and after 100 ps, the system stabilizes.

**Figure 3:**
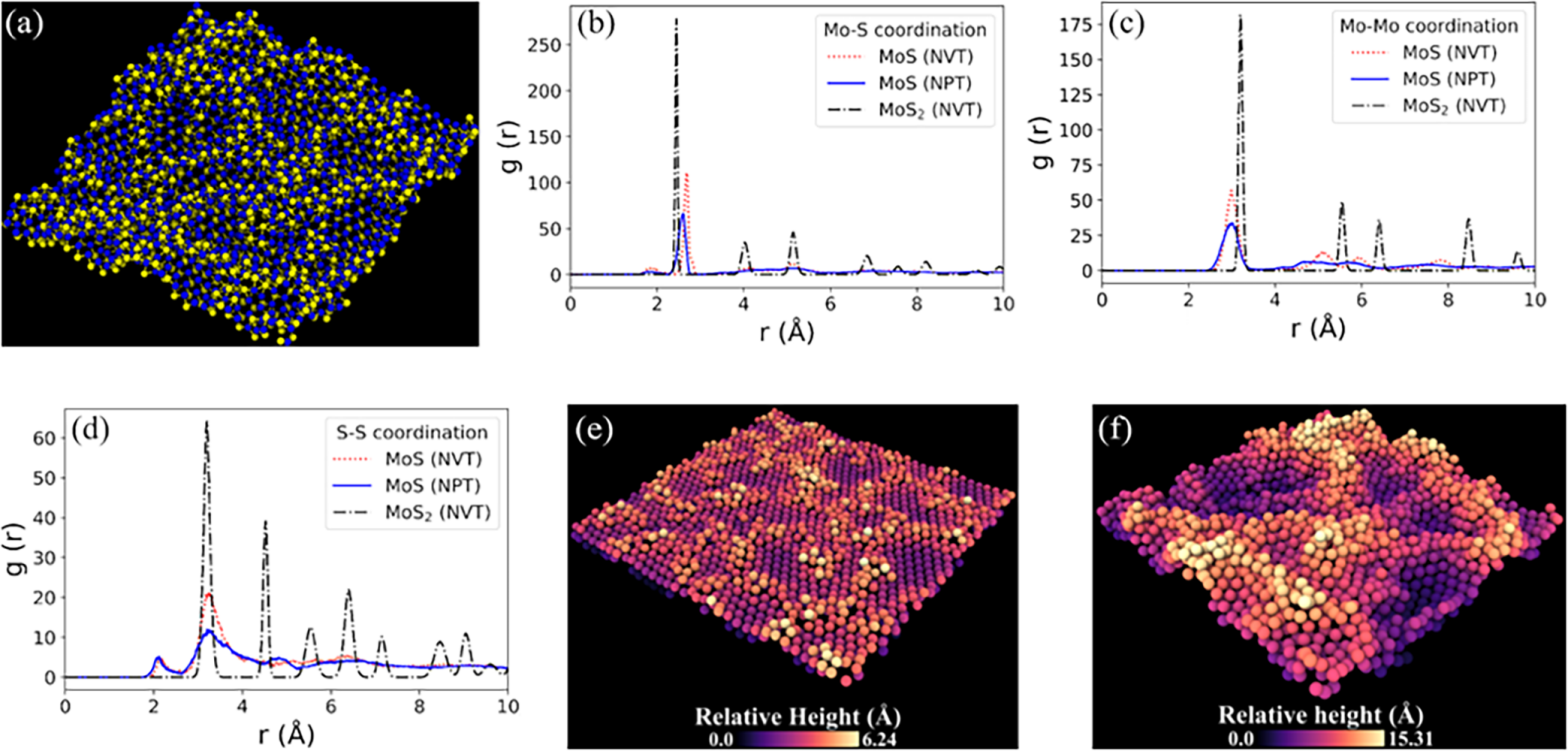
(a) Equilibrated MoS configuration after 100 ps of CMD in the NPT ensemble. Coordination analyses were performed between (b) Mo–S, (c) Mo–Mo, and (d) S–S for MoS and MoS_2_ monolayers. Relative-height mapping of MoS after equilibration in (e) NVT and (f) NPT ensembles.

Building on these population trends, the spatial organization of the reconstructed lattice reveals how sulfur migration translates into nanometric structural domains. In the 300 K NVT configuration, Figure 1b,d shows that adjacent S-dislocation lines are typically separated by ∼4 to 8 lattice periods, yielding a lateral domain width of *L*_dom_ ≈ 1.3–2.5 nm, with occasional contiguous patches spanning ∼3 to 4 nm. Under NPT, Figure 3a reveals that the early in-plane contraction produces slightly tighter mosaics, reduced by roughly 10–20% relative to NVT while preserving the same line-like topology. The relative-height maps in Figure 3e,f corroborate this behavior: the corrugation amplitude ranges from ∼0 to 5.2 Å in NVT to ∼0 to 15.3 Å in NPT, and the wavelength of the height undulations is λ ≈ 2–4 nm, consistent with the inferred in-plane domain widths. Overall, the reconstruction consists of nanometric domains (median ∼1.5 to 2 nm at 300 K, NVT), a slight decrease under NPT due to lateral relaxation, and a robust morphology defined by line-like S dislocations that partition MoS-coordinated patches rather than restoring a fully sulfurated sheet.

Figure 3b also presents the coordination of Mo–S atoms in both MoS and MoS_2_ structures. The first peak in the coordination function for MoS_2_ is well-defined, at approximately 0.243 nm. In contrast, this distance exhibits a broader distribution in MoS (for both NVT and NPT ensembles). This broadening occurs in association with changes in Mo–Mo coordination, as depicted in Figure 3c. In the case of MoS, the Mo–Mo coordination pattern widens and displays nearest-neighbor peaks smaller than those in pristine MoS_2_. This phenomenon results from sulfur depletion on the surface, leading to a decrease in the Mo–Mo distance. This reduced distance allows sulfur atoms to migrate across the surface at locations where adjacent Mo atoms are separated. As sulfur atoms in that region move toward the depleted area (L1), the adjoining parts expand, promoting the migration of more sulfur atoms across the Mo layer.

As the system evolves, equilibrium is achieved when a sufficient number of S atoms migrate across the surface and form Mo clusters. This process leads to a substantial change in the S–S coordination in MoS (see Figure 3d), contrasting with the behavior in MoS_2_. Notably, boundary lines of S atoms emerge when they transverse the Mo layer, resulting in an S–S coordination of approximately 0.2 nm. The S–S coordination widens for the S atoms that remain in the L3 layer compared to MoS_2_. However, the peak location remains roughly the same.

It was also observed that during the NVT equilibration, the monolayer undergoes significant changes, resulting in a corrugated surface with a thickness of approximately 0.624 nm (see Figure 3e). This corrugation becomes even more pronounced when the system is switched to the NPT ensemble, as is evident in the height map shown in Figure 3f. Moreover, this trend suggests that, despite achieving stability in terms of constant area (in the NVT ensemble), the extent of S depletion is substantial enough for the system to continue striving to lower its energy by reducing the overall distance between atoms.

Guided by the images in Figures 1b–d and 3a,e,f, the in-plane spacing between neighboring S-dislocation lines at 300 K (NVT) is on the order of *L*_dom_ ≈ 1.3–2.5 nm, with contiguous patches occasionally reaching ∼3 to 4 nm. This length is consistent with the practical threshold we report for supported sheets, where traversal is suppressed for minimal defective regions but emerges once the depleted area exceeds ∼4 nm^2^ (lateral scale ∼2 nm). Under NPT, early lateral relaxation produces a slight decrease of the mosaic size (by ∼10 to 20%). Still, the characteristic length remains in the same nanometric range, in agreement with the relative-height maps (Figure 3e,f), whose undulation wavelength is also ∼2 to 4 nm.

Both NVT and NPT simulations exhibit out-of-plane corrugation with larger amplitudes under NPT conditions. This corrugation facilitates the early stages of reconstruction by locally relieving in-plane strain and transiently lowering the energetic cost for sulfur atoms to cross the Mo plane. Regions of positive curvature promote reduced Mo–S coordination and bond stretching on the depleted side, favoring the first traversal event that nucleates reconstruction. As domains form, corrugation becomes partially accommodated within the dislocation network so that subsequent sulfur migration is governed primarily by in-plane strain rather than by further increases in bending amplitude.

Until now, our investigation has focused on removing one layer of sulfur atoms from a suspended MoS_2_ monolayer. In experimental settings, TMDs are typically synthesized on substrates, and this interaction can significantly alter the material’s dynamics. The presence of a substrate restrains movement normal to the surface. It also introduces surface interactions, both of which can be fundamental in determining the final morphology of the defective TMDs.

To investigate these substrate effects, we carried out additional simulations using systems where our MoS monolayer was placed atop pristine MoS_2_, effectively mimicking the role of a substrate. We systematically varied the size of the defect area in these simulations, as illustrated in Figure 4.

**Figure 4:**
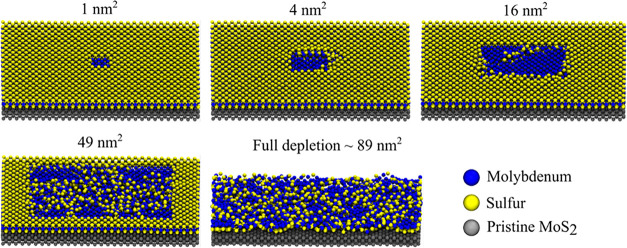
Equilibrated configurations after NPT runs for systems with varying defect sizes, ranging from 1 to 89 nm^2^ on top of a pristine MoS_2_ substrate.

For the smallest defect area considered here (1 nm^2^), we have observed an intriguing phenomenon where no sulfur atoms from L3 move to L1. The depletion of sulfur atoms caused the Mo atoms in the defect area to approach each other, resulting in a local shrinkage of the structure. This process formed a slight indentation on the surface, preventing the sulfur atoms underneath from migrating to the L1 site. This phenomenon bears similarities to the effect of chalcogenide substitution in Janus structures, where, for instance, selenium atoms replacing sulfur on one side of MoS_2_ can lead to the formation of scrolls and/or nanotubes, as demonstrated in ref 47,47.

Regarding a defect area of 4 nm^2^, we have observed a similar effect where the local contraction of Mo atoms caused the boundary between MoS and MoS_2_ to move farther apart. This expansion provided sufficient space for S atoms to migrate from L3 to L1. Notably, this phenomenon was also observed as the defect area increased (49 and 89 nm^2^). As the defect area grew, it increasingly resembled the total S removal shown in Figure 1. The appearance of complete S depletion on the top of the MoS_2_ substrate resembles that of suspended MoS. However, the relative height of MoS in this substrate-supported scenario, measured at 5.77 Å after NPT equilibration, was even smaller than that in the suspended case during NVT equilibration. This trend indicates that a substrate has a flattening effect on the MoS surface due to interlayer interactions. Nevertheless, the dynamics of S atoms moving from L3 to L1 remained consistent if the defect area was sufficiently large (approximately 4 nm^2^). Overall, small defective regions (compact areas in the few-nm^2^ range) visibly suppress traversal or confine it locally. Beyond that scale, the behavior approaches that of larger/suspended sheets with extended S lines and broader repopulation on the depleted side.

Direct (S)­TEM studies on monolayer MoS_2_ have imaged atomic diffusion and the evolution of vacancies/dislocations, consistent with thermally activated migration and line-like reconstructions observed here.^48,49^ Selective top-layer chalcogen replacement yielding Janus MoSSe has been realized (including room-temperature atomic-layer substitution), supporting the kind of one-sided repopulation/asymmetry we obtained in Figures 1–3.^50,51^ Temperature-controlled bilayer growth further reveals AA/AB stacking domains and registry changes, consistent with our substrate-assisted high-*T* registry islands.^52,53^ Finally, defect-engineered MoS_2_ films exhibit enhanced HER activity, consistent with an increased density of edge-/defect-like motifs discussed in our work.^32^

Although explicit electronic calculations are beyond the scope of this work, the reconstructed motifs observed here are expected to impact the local electronic landscape. Line-like S dislocations and Mo-rich patches introduce under- and mis-coordinated sites that can locally modify band alignment and facilitate charge transfer, while AA/AB registry islands formed on the substrate may induce variations in interlayer coupling and local density of states. Such effects are consistent with experimentally reported changes in the electronic and catalytic behavior in defect-engineered MoS_2_ systems.

Partial S traversal generates a mosaic of MoS_*x*_ domains separated by line-like S dislocations, while higher temperatures promote Mo adsorption and the formation of local AA/AB registry islands on the support. These reconstructed motifs are expected to alter the electronic landscape and, thus, the HER response relative to a clean MoS surface. Dislocation lines behave as extended edge- or vacancy-like features; therefore, increasing the migrated fraction (*f*_mig_) or decreasing the lateral domain width (*L*_dom_) enhances the density of under- and mis-coordinated sites that typically strengthen H adsorption and facilitate charge transfer. Repopulating only the top chalcogen layer introduces local Janus-like asymmetry, dipoles, and band offsets. In contrast, strong interlayer coupling within the AA/AB islands at higher temperatures can locally metalize or passivate regions. Taken together, the images suggest a nonmonotonic trend: as *f*_mig_ increases from low to moderate values and domains become smaller, activity should initially rise; at still higher temperatures or under strong substrate coupling, Mo adsorption and registry locking reduce the number of accessible active motifs and may suppress activity.

In our simulations carried out at 300 K, we observed single Mo–S bonds and no bonding between the Mo atoms from MoS and the S atoms of the pristine MoS_2_ substrate. However, to explore how this system behaves at elevated temperatures, we carried out further simulations of the MoS/MoS_2_ system at temperatures of 600, 800, and 1000 K. Upon increasing the temperature to 600 K, we observed an increase in bonding between the Mo atoms from MoS and the S atoms of MoS_2_. This phenomenon arises because the Mo atoms in the MoS layer, where S migrated from L3 to L1, become more mobile. Higher temperatures provide them the energy required to overcome the thermal barriers and to interact with the S atoms from MoS_2_.

As the temperature increases, the number of Mo atoms from MoS that are adsorbed onto MoS_2_ also increases, as illustrated in Figure 5a. Higher temperatures accelerate the process of Mo adsorption, resulting in the faster degradation of the MoS layer. However, up to the 2 ns time scale, the system had not yet reached complete adsorption. Another interesting fact is that the coordination of Mo atoms added to the pristine MoS_2_ structure follows AB and AA stacking formations along the surface. The regions are clustered in either of the stacked configurations, as some Mo atoms are positioned on the top of S atoms.

**Figure 5:**
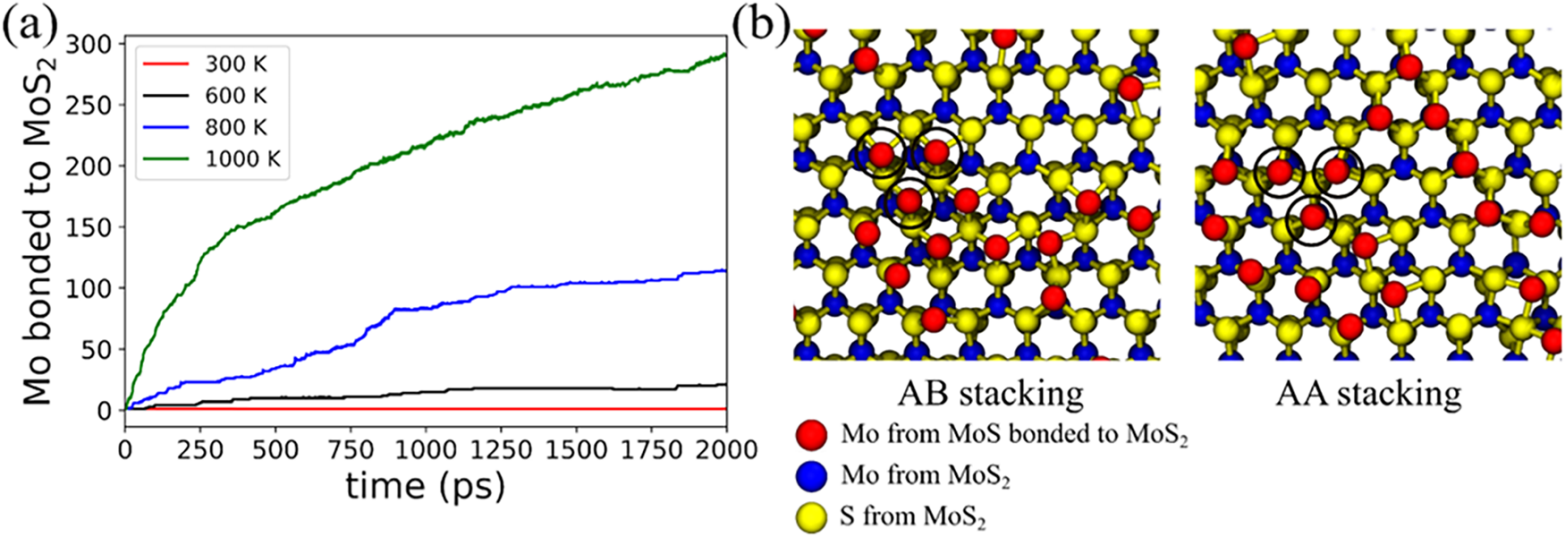
(a) The number of Mo atoms from MoS added to the MoS_2_ layer. (b) CMD snapshots showing AB and AA stacking configurations formed between pristine MoS_2_ and Mo from MoS after 2 ns of equilibration at 1000 K.

Placing our results in a broader context, defect-mediated stabilization is a recurrent feature in 2D materials.^54^ In chalcogen-deficient transition metal dichalcogenides such as MoS_2–*x*_, WS_2–*x*_, and MoSe_2–*x*_, thermal treatments between 600 and 1000 K promote chalcogen desorption, adatom and vacancy diffusion, and chalcogen exchange, enabling the formation of Janus and alloyed phases such as MoSSe and MoS_*x*_Se_2–*x*_.^21,50,55,56^ Related effects occur in two-dimensional oxides such as MoO_3–*x*_ and TiO_2–*x*_, where anion vacancies migrate and heal upon annealing.^57^ In contrast, in graphene and h-BN, vacancies tend to reconstruct through bond rotations and nonhexagonal ring formation rather than cross-plane motion.^58,59^ Within this broader framework, our observation that sulfur atoms in MoS traverse the Mo plane to repopulate a depleted surface reveals an atomistic pathway for stabilizing chalcogen-depleted TMD layers and forming MoS_*x*_ patches *in situ*. Since chalcogen bond strengths and diffusion barriers scale across group-VI TMDs, similar migration and replenishment mechanisms are expected in WS_2_, MoSe_2_, and WSe_2_, with temperature ranges shifted according to their bond energetics.^22,60^ In the presence of a chalcogen reservoir such as selenium vapor or a Se-terminated substrate, cross-plane traversal may favor Janus-like or alloyed configurations.^50,61^ The substrate-assisted Mo adsorption observed between 600 and 1000 K is likely a general feature whenever a chalcogen-depleted overlayer rests on a pristine TMD, facilitating AA or AB registry domains, interfacial alloying, and gradual degradation of the overlayer. The practical size threshold identified for sustained sulfur migration, approximately 4 nm^2^ for supported MoS, suggests that the defect area is a key parameter governing the onset or suppression of traversal in other chalcogen-depleted TMDs.

## 4 Conclusions

In summary, our study has revealed the remarkable stability of monolayer MoS_2_ structures with one complete sulfur layer removed, aptly referred to as MoS. After removing all sulfur atoms from the top layer, several sulfur atoms from the bottom layer spontaneously migrate to the top layer as a response to increase structural stability, thus creating a MoS_*cx*_ alloy. Our findings are based on consistent results obtained through two distinct methodologies, AIMD and reactive CMD. Suspended and supported MoS monolayers exhibit stability over large areas, forming extensive regions where the initial Mo–S structure remains intact. This stability, however, comes at the cost of some sulfur atom dislocation in the depleted region of the monolayer.

Our simulations unveil an interesting temperature-dependent phenomenon in the context of supported MoS monolayers. At elevated temperatures, Mo atoms from MoS bond with the underlying pristine MoS_2_ layer, forming islands with AB or AA stacking configurations. These insights into atomic manipulation within TMDs offer a promising avenue for creating novel morphologies and synthesizing Janus structures, paving the way for innovative applications of these compounds in nanoscience and technology. This investigation enhances our understanding of defect dynamics in TMDs and provides practical approaches to engineering their properties for diverse nanotechnology applications.

The findings also suggest that several processes can bias sulfur traversal without changing the underlying mechanism. In-plane strain is primary: tensile strain on the depleted side tends to facilitate traversal and shrink domains, whereas compression suppresses it (in line with the smaller mosaics under NPT). Controlled curvature/bending (wrinkles or patterned relief) can direct the traversal toward the convex side and orient S lines. Substrate adhesion/registry also matters: strongly binding or reactive supports promote cross-interface Mo bonding at high temperature and arrest further S accumulation, while weak van der Waals supports (e.g., graphene, h-BN) preserve larger MoS-like patches and higher migrated fractions. Finally, the chalcogen chemical potential (background S/Se flux), gentle thermal ramps, and out-of-plane electrostatic bias provide additional mechanisms to adjust the saturation fraction and control the domain size.

## Supplementary Material







## Notes

Molecular dynamics simulation showing the sulfur depletion process in defective monolayer MoS_2_ (full view) (Video 1) (MP4)
Zoomed-in molecular dynamics view of sulfur depletion in defective monolayer MoS_2_ (Video 2) (MP4)
Lateral zoomed-in view from molecular dynamics simulations illustrating sulfur depletion in defective monolayer MoS_2_ (Video 3) (MP4)
